# Symptom Clusters in Middle Age and Older Adults Prior to Treatment for Cancer

**DOI:** 10.1093/geroni/igaf122.3209

**Published:** 2025-12-31

**Authors:** Victoria Loerzel, Kailei Yan

**Affiliations:** University of Central Florida, Orlando, Florida, United States; University of Central Florida, Orlando, Florida, United States

## Abstract

Health at the beginning of cancer treatment is becoming more complex. While much of the focus on symptoms is centered on during treatment, many adults start treatment for cancer with multiple symptoms related to their diagnosis and other co-morbid illnesses due to age. Identifying symptom clusters (SC) prior to treatment is critical so they can be managed, and we can assist aging adults in maintaining well-being and quality of life. The goal of this study was to identify symptom clusters in middle age and older adults prior to being enrolled in an RCT for symptom management. We used exploratory factor analysis to extract symptom clusters, and model fit indices were evaluated. Then, the same method was applied to analyze subgroup difference between older (aged at least 65) and middle-aged (>50, < 65) participants. 141 adults aged 50 and over were included in analysis. The 14-item Symptom Representation Questionnaire was used to report symptoms. Participants aged 65 and older had 2 distinct SC: Physical and Cognitive/Emotional. Participants aged 64 and younger had 3 SC with unsimilar and difficult to categorize symptoms: Emotional/Neurological, Physical, and Miscellaneous. Each cluster had between 4 and 8 individual symptoms and the Physical clusters were not identical. SC appears to differ by age as does the clarity of the cluster. Assessing SC prior to treatment may lead to early awareness, symptom self-management education, and prevention of worsening symptoms and quality of life in these complex adults.

